# Cognitive Reserve as a Predictor of Age of Onset and Cognitive Decline in Patients with Alzheimer’s Disease

**DOI:** 10.1002/alz.088255

**Published:** 2025-01-09

**Authors:** Anat Marmor, Eli Vakil, Shlomzion Kahana Merhavi, Zeev Meiner

**Affiliations:** ^1^ Hadassah Hebrew University Medical Center, Jerusalem Israel; ^2^ Bar Ilan University, Jerusalem Israel

## Abstract

**Background:**

The cognitive reserve (CR) theory seeks to explain the mismatch often reported between brain damage and its clinical expression. Unlike most previous studies that focused on individuals with memory deterioration before the diagnosis of Alzheimer’s disease (AD), the present study examined the late stages of the disease. The study sought to confirm the hypothesis that patients with higher CR are diagnosed later and decline faster than those with lower CR because their brain pathology is more severe.

**Method:**

This is a retrospective study conducted at a memory clinic and included 642 (361 female) individuals above the age of 65 diagnosed with AD. Average age was 77.01 (range = 65‐97). Cognitive decline was evaluated using the Mini Mental State Examination (MMSE).

**Result:**

In the entire sample, no significant correlation was found between the age of diagnosis and CR. Therefore, the sample was divided into two groups, according to years of education: (a) patients with nine years of high education or more (n = 442, with average years of education 13.93, SD = 3.00) and (b) patients with eight years of education or less (n = 141, with average years of education 5.21, SD = 3.08). Our results show that only in the group of patients with higher education, education level was significantly associated with delayed age of diagnosis. As predicted, more years of education were associated with steeper cognitive decline in both groups.

**Conclusion:**

The study examined the less studied group of individuals at late stages of AD. The study shows that the effect of education on the age of diagnosis is not linear and it is valid for those with a higher but not necessarily for those with lower education. In patients with higher education, higher CR resulted in delayed age of diagnosis but also faster deterioration; in the lower education group, the resilience factor that leads to later diagnosis is not present. The implications of the study include diagnostic and therapeutic guidelines that match the different characteristics of each group of patients.

